# Hemodynamic collapse induced by general anesthesia in a patient with an unruptured thoracic aortic aneurysm: a case report

**DOI:** 10.1186/1471-2261-13-122

**Published:** 2013-12-27

**Authors:** Oto Inoue, Hisayoshi Murai, Shuichi Kaneko, Soichiro Usui, Hiroshi Furusho, Masayuki Takamura

**Affiliations:** 1 Department of Disease Control and Homeostasis, Graduate School of Medical Science, Kanazawa University, 13-1 Takara-machi, Kanazawa 920-8641, Japan

**Keywords:** Thoracic aortic aneurysm, Anesthesia, Percutaneous cardiopulmonary support

## Abstract

**Background:**

Compression of the trachea, bronchi, and pulmonary arteries are complications in patients with large thoracic aortic aneurysms. In this case, we report unexpected cardiopulmonary collapse manifested by general anesthesia before surgery in an asymptomatic patient with a large thoracic aortic aneurysm.

**Case presentation:**

We present the case of a 32-year-old man with a 10-cm aneurysm in the ascending aorta. A total aortic arch replacement was planned. After intravenous anesthesia, his aneurysm occluded the left main bronchus and right pulmonary artery simultaneously, and induced severe hypoxia. Percutaneous cardiopulmonary support was conducted and the patient recovered from cardiopulmonary collapse successfully. After the patient regained consciousness from anesthesia, the findings of organ compressions disappeared. At the second surgery, percutaneous cardiopulmonary support was initiated with local anesthesia before general anesthesia and intubation. The operation was performed successfully without any adverse events.

**Conclusion:**

We experienced a case of hemodynamic collapse induced by general anesthesia in a patient of an unruptured thoracic aortic aneurysm. It is important to recognize that fatal organ compression might be caused by general anesthesia even in asymptomatic patients with thoracic aortic aneurysm.

## Background

Compression of the trachea, bronchi, and pulmonary arteries are complications in patients with large thoracic aortic aneurysms (TAAs) [1]. Several reports have indicated that a large aneurysm contributes to severe hypoxia or hemodynamic collapse without resulting in rupture or dissection of the aneurysm. However, in each case, the patient complained of dyspnea upon waking or while in the supine position. We report unexpected cardiopulmonary collapse manifested by general anesthesia before surgery in an asymptomatic patient with a large TAA.

## Case presentation

**Figure 1:**
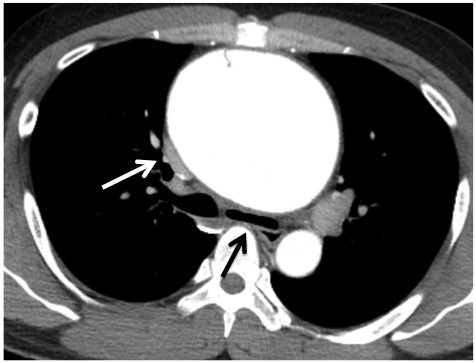
**Preoperative chest CT.** CT scan showing a large, thoracic aortic aneurysm (TAA) in the ascending aorta with a small aortic dissection that did not reach the sinus of Valsalva. The descending aorta was intact. There were no signs of rupture, such as pericardial effusion or hemothorax. The right pulmonary artery (white arrow) and the left main bronchus (black arrow) was compressed but not occluded.

A 32-year-old male presented to the outpatient division of our hospital with coughing and a slight fever. He had a history of aortic valve stenosis (bicuspid valve) and reduced LV ejection fraction (EF, 40%) at the age of 5 years. He had received an operation of aortic valve replacement at the age of 16 years. After surgery, he was followed up by another hospital and was given warfarin with fair control. At room temperature, the patient’s physical parameters were as follows: temperature, 37.0°C; blood pressure, 90/60 mmHg; pulse, 89 beats/min; and oxygen saturation, 99% (room air). Auscultation revealed normal breath sounds. A grade II/VI systolic ejection murmur was noted at the right sternal border. A chest radiograph revealed marked cardiomegaly and mediastinum expansion without a difference of increased radiolucency between the left and right lungs. A chest computed tomography (CT) scan revealed a 10-cm aneurysm in the ascending aorta (Figure 1). The CT image indicated that the TAA had compressed the right pulmonary artery without any difference of contrast medium character between left and right lung, indicating that the right pulmonary artery blood flow was not disturbed. An echocardiogram showed left ventricular (LV) enlargement (LV end-diastolic diameter, 57 mm) and diffuse LV hypokinesis (EF, 35%). A Doppler echocardiogram showed a trans-aortic valve pressure gradient of 18 mmHg. However, his exercise tolerance was well. Any dyspnea on effort had not seen during out-patient clinic (NYHA I). Ventilation/perfusion lung scintigraphy was conducted to exclude aneurysm-induced compression of surrounding organs, but no significant findings were observed. The patient had never complained of dyspnea during nighttime in the supine position either.

**Figure 2:**
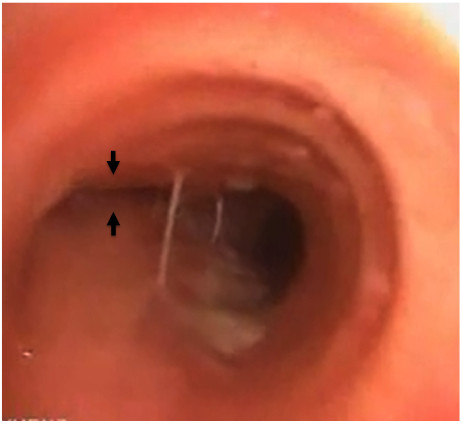
**Bronchoscopy after intubation.** Bronchoscopy at the carina shows the totally occluded ostium of the left main bronchus (black arrow).

A total aortic arch replacement was planned. However, after intravenous anesthesia (his body weight 78 kg, 5-mg midazolam, 100-mg thiopental and a total of 90-mg rocuronium) and intubation in the supine position, his oxygen saturation (70%) and blood pressure (60/40 mmHg) decreased rapidly. His arterial gas analysis showed the following results on FiO_2_ 100%: pH, 7.234; PaO_2_, 20 mmHg; PaCO_2_, 72.6 mmHg; SaO_2_, 18.6% and HCO_3_^–^, 29.6 mmol/L. Cardiopulmonary collapse was resistant to vasopressors (total 12-mg ephedrine) and high oxygenation. As acute pulmonary embolism was tentatively considered in this patient, pulmonary arteriography was immediately performed. The left pulmonary artery was patent. However the right pulmonary artery was totally defected. Swan–Ganz catheter was unable to pass the main branch of the right pulmonary artery. Additionally, because of the absence of breath sounds on auscultation in the left lung field, bronchoscopy was performed and complete compression of the left main bronchus was noted (Figure 2). We finally diagnosed sudden hemodynamic collapse and hypoxia as simultaneous compression of both right pulmonary artery and left main bronchus.

**Figure 3:**
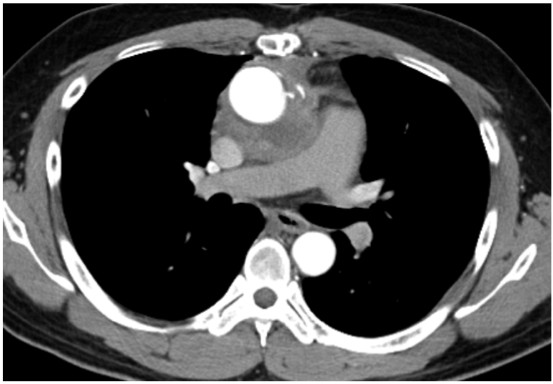
**Postoperative chest CT.** No compressions were observed at the left main bronchus and the right pulmonary artery after total aortic arch replacement.

Percutaneous cardiopulmonary support (PCPS) was conducted resulting in recover from the cardiopulmonary collapse effectively. We discontinued the operation and transferred the patient to the intensive care unit. After the patient regained consciousness from anesthesia, his hemodynamic collapse was gradually improved, and weaning of PCPS was achieved without use of inotropes. Besides, bronchoscopy revealed that occlusion of the left main bronchus disappeared, and he was successfully extubated. Fortunately, he recovered the next day without neurological deficit. At the second surgery, PCPS was initiated with local anesthesia before general anesthesia and intubation. We denuded the right femoral vein and artery, and introduced cannulas for PCPS. General anesthesia was conducted carefully. We also performed bronchoscopy after intubation and found out that the left main bronchus was almost occluded in much the same way as the prior operation. However, his oxygen saturation was not decreased. At this time, PCPS was thought to be useful to prevent from hypoxia and hemodynamic collapse. The aortic valve prosthesis that previously replaced was completely intact. The aortic aneurysm occupied from just above the prosthesis to the distal aortic arch. We successfully performed total arch replacement and reconstruction of bilateral coronary arteries, brachiocephalic artery, left common carotid artery and left subclavian artery without any adverse events (Figure 3).

## Discussion

Large TAAs are known to compress surrounding organs [1]. However, this is the first report of an unexpected hemodynamic collapse induced by general anesthesia in an asymptomatic patient with a large TAA. In our case, the bronchus and pulmonary artery were totally compressed after induction of general anesthesia, which might have contributed to the ventilation-perfusion mismatch; reduction in the right pulmonary blood flow and the left pulmonary ventilation. Interestingly, the compression disappeared as the anesthetic wore off.

Because of the closed anatomical arrangement and limited space in the thoracic cavity, the trachea, bronchi, and pulmonary arteries are sandwiched between the aortic aneurysm and the vertebral column. Two plausible explanations responsible for the cardiopulmonary collapse by general anesthesia were considered in this patient. One is that general anesthesia and/or muscle relaxants weaken respiratory muscles (such as the intercostal muscles) that contribute to a reduction in the thoracic cavity and inspiratory rib elevation [2,3]. The other is that positive pressure ventilation per se directly elevated the intrapleural pressure, which might have compressed mediastinum organs including aneurysm, pulmonary artery and bronchus. Conceivable scenario in our case is assumed as follow; firstly the large aneurysm occluded the left main bronchus due to sedative drugs, secondly the right thoracic cavity gained air volume because of the reduction in left side lung volume, and finally the right pulmonary artery was occluded with aneurysm as well as thoracic pressure with expanded right lung volume.

In this case, LV systolic dysfunction was detected in echocardiography before surgery. There is a possibility that cardiogenic shock might have been involved in the hemodynamic collapse. However, pulmonary artery pressure was not elevated (systolic/diastolic pressure; 30/15 mmHg) in Swan-Ganz catheter measurements, suggesting no secondary pulmonary hypertension. Besides, chest X-ray and electrocardiogram showed no significant findings. Taken together, it is unlikely that sudden hemodynamic collapse was associated with poor LV function.

Two cases of TAA-induced severe hypoxia with compression of the left main bronchus and right pulmonary artery have been reported [4,5]. These differ from our case in terms of symptoms. Sugimoto *et al*. reported a patient with a large TAA, who had dyspnea particularly in the supine position, but underwent successful surgery. Preoperative symptoms seemed to be useful to treat the patient using tracheal intubation and extracorporeal bypass from the femoral vein to the artery in the half-sitting position. Türköz *et al*. reported acute hemodynamic collapse in a patient who had dyspnea at rest that emerged in the supine position without the use of any drug. Emergency surgery was performed, but was not successful. In our case, the symptoms in terms of compression were not manifested by the supine position but appeared after induction of anesthesia. We elected to establish PCPS to maintain oxygenation before induction of general anesthesia. The operation was successful and the patient’s postoperative course was uneventful.

From our case, careful inspections in preoperative CT scan are thought to be significant in order to predict hemodynamic collapse. Because, the compression (not occlusion) of the left bronchus and the right pulmonary artery were already observed in preoperative chest CT scan as shown in Figure 1. Retrospectively, it might have been the only clue to predict the severe cardiopulmonary event. Surprisingly, the left main bronchus was occluded again in the second surgery. Sudden onset of hemodynamic collapse during surgery often brings about critical course because of the difficulty in diagnosis. For this reason, it is important to recognize that fatal organ compression might be caused by general anesthesia in asymptomatic patients with TAA in which organ compression is slightly seen. In such case, the establishment of PCPS before induction of general anesthesia is one of the most reliable choices to prevent hemodynamic collapse.

## Conclusions

In summary, we successfully treated a patient with a large TAA who presented with left main bronchus and right pulmonary artery occlusions by establishing PCPS before induction of general anesthesia and intubation. The preoperative CT scan showed that the right pulmonary artery and the left main bronchus was compressed but not occluded. However, it might be useful for predicting the severe cardiopulmonary collapse. We emphasize that it is crucial to recognize that fatal organ compression might be caused by general anesthesia in asymptomatic patients with TAA.

## Consent

Written informed consent was obtained from the patient for publication of this case report and any accompanying images. A copy of the written consent is available for review by the Editor-in-Chief of this journal.

## Abbreviations

CT: Computed tomography; LV: Left ventricle; PCPS: Percutaneous cardiopulmonary support; TAA: Thoracic aortic aneurysm.

## Competing interests

The authors declare that they have no competing interests.

## Authors’ contributions

OI, HM and MT have done the patient’s follow-up and wrote the manuscript. HM, SU and HF conducted the clinical diagnosis and data collection. SK involved in drafting the manuscript and revising it critically for important content. All authors read and approved the final manuscript.

## Pre-publication history

The pre-publication history for this paper can be accessed here:

http://www.biomedcentral.com/1471-2261/13/122/prepub
